# Does a mobile application lend a hand for promoting antimicrobial stewardship? A survey of users

**DOI:** 10.1017/ash.2026.10813

**Published:** 2026-09-03

**Authors:** Nicole Le Saux, Jennifer Bowes, Anne Tsampalieros, Cora Constantinescu, Carsten Krueger, Waleed Alqurashi

**Affiliations:** 1 Infectious Diseases, Children’s Hospital of Eastern Ontario, Ottawa, Canada; 2 Children’s Hospital of Eastern Ontario Research Institute, Ottawa, Canada; 3 Infectious Diseases, Alberta Children’s Hospital, Calgary, Canada; 4 Emergency Medicine, Children’s Hospital of Eastern Ontario, Ottawa, Canada

## Abstract

Analytics of antimicrobial stewardship applications (ASApp) from two pediatric hospitals over 24 months revealed an average of 4922 monthly sessions. A survey of users reported use in all clinical areas with 30% exclusively in outpatient settings. Overall, >70% reported using the recommendations and almost all self-reported an impact on practice.

## Graphical abstract

**Figure:**
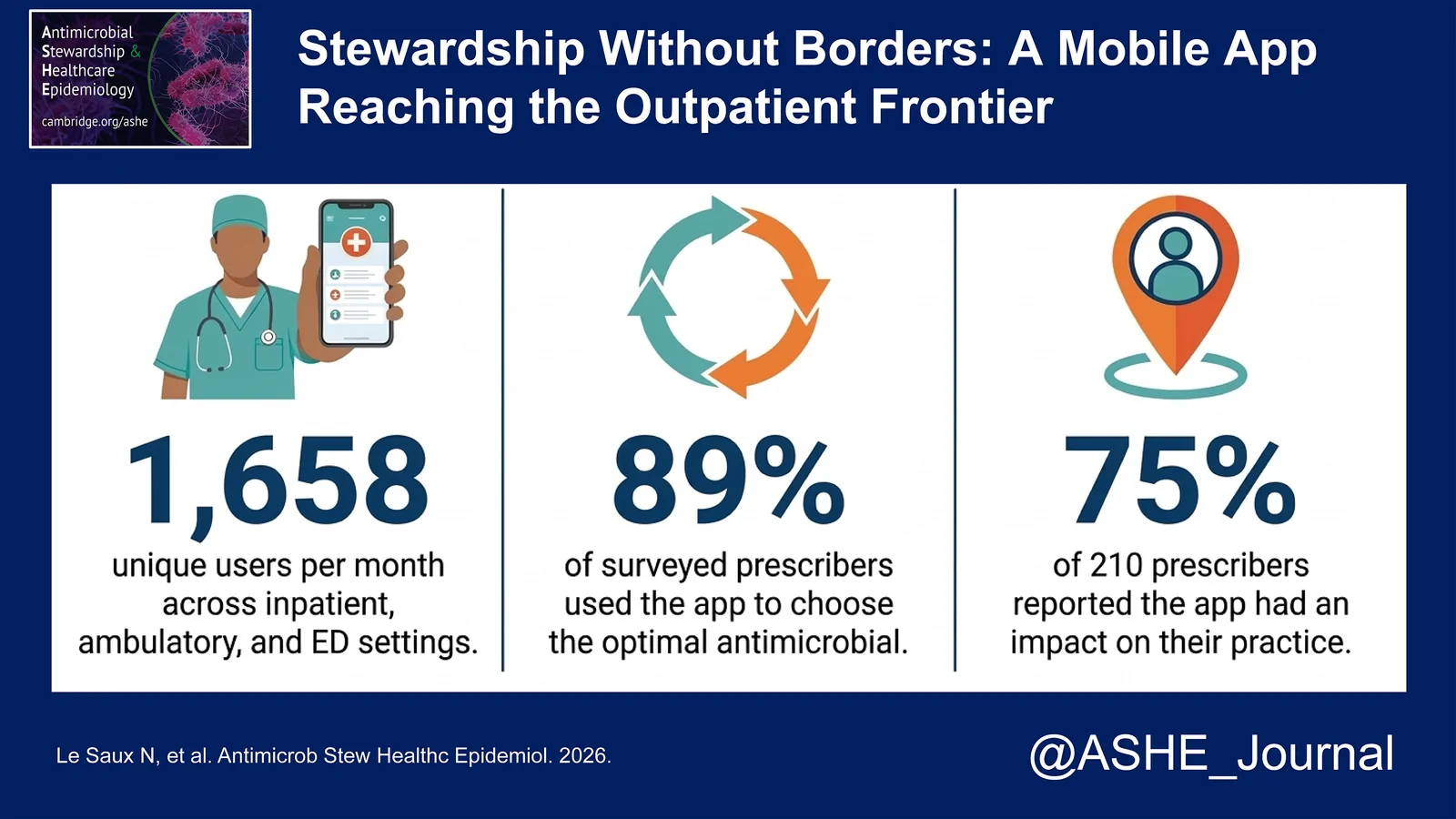

## Background

Prospective audit and feedback is an effective strategy for antimicrobial stewardship (AS), but challenges include time, and human resources. Expansion of AS tools to support self-stewardship are potential solutions to disseminate guidance and influence practice.^1^

The extent of use of an ASApp and whether users find it helpful for pediatric practice across the spectrum of health care is not well-known. The primary objective of this study was to determine characteristics of use of an ASApp and, through a survey, determine whether users reported using the information provided and if the ASApp had impacted their practice.

## Methods

An ASApp (Firstline™, formerly Spectrum™) was introduced separately at two pediatric hospitals (Children’s Hospital of Eastern Ontario [CHEO], in Ottawa, Ontario and Alberta Children’s Hospital [ACH] in Calgary, Alberta) in 2017. The two sites are Canadian tertiary care referral centers in different provinces and are the only pediatric hospitals in their respective catchment areas. Content in both ASApps include common infectious syndromes however, each site provides recommendations independently based on local practice and resistance patterns. Sections include diagnostic/clinical considerations, and recommendations for choice, dose and duration of antimicrobials with content aimed at promoting AS. The ASApp is free, accessible to any end-user and can be downloaded on a mobile phone or computer. The ASApp is not linked to patient medical records so prescriptions for dispensed medication cannot be obtained.

ASApp analytics (number of unique users, sessions and most accessed guidelines) were obtained from Firstline™. The number of monthly ASApp users is the number of unique individuals that have accessed the application at least once in a calendar month. One session is defined as 1 or more uses within a 30-minute period.

The survey adhered to the Consensus-based Checklist for Reporting of Survey Studies methodology.^2^ Survey questions were developed within specific themes with corresponding questions: users (questions a,b,c,d), use of recommendations (e), education (f,g), and impact on clinical practice (h). (Appendix 1) The survey tool was piloted among 10 hospital-based pediatricians with their minor feedback incorporated. A direct link to the on-line survey was visible upon opening the ASApp. The survey was conducted using REDCap (version: 16.0.13 Vanderbilt University, Nashville, TN) with responses transmitted directly to the research site and not linked to the analytics. The survey was available from April 29 to July 14, 2025 at CHEO and May 6, 2025 to August 1, 2025 at ACH. After 2 weeks, reminder emails were sent to physicians at both hospitals and posters with a QR code link were displayed. Only one response per email address was allowed. Research Ethics approval was obtained at both sites prior to initiation of the survey.

Descriptive statistics were conducted using R statistical programming language (Version 4.4.1).^3^ Characteristics of the respondents and responses to questions were summarized using frequencies and percentages.

## Results

Between December 1, 2023 and November 30, 2025, there was a monthly average of 1658 unique users and 4,922 sessions at both sites. The most frequently accessed guidelines (in descending order) were acute otitis media, community acquired pneumonia, acute cellulitis, pharyngitis, febrile urinary tract infection and neonatal sepsis.

**Figure 1.**
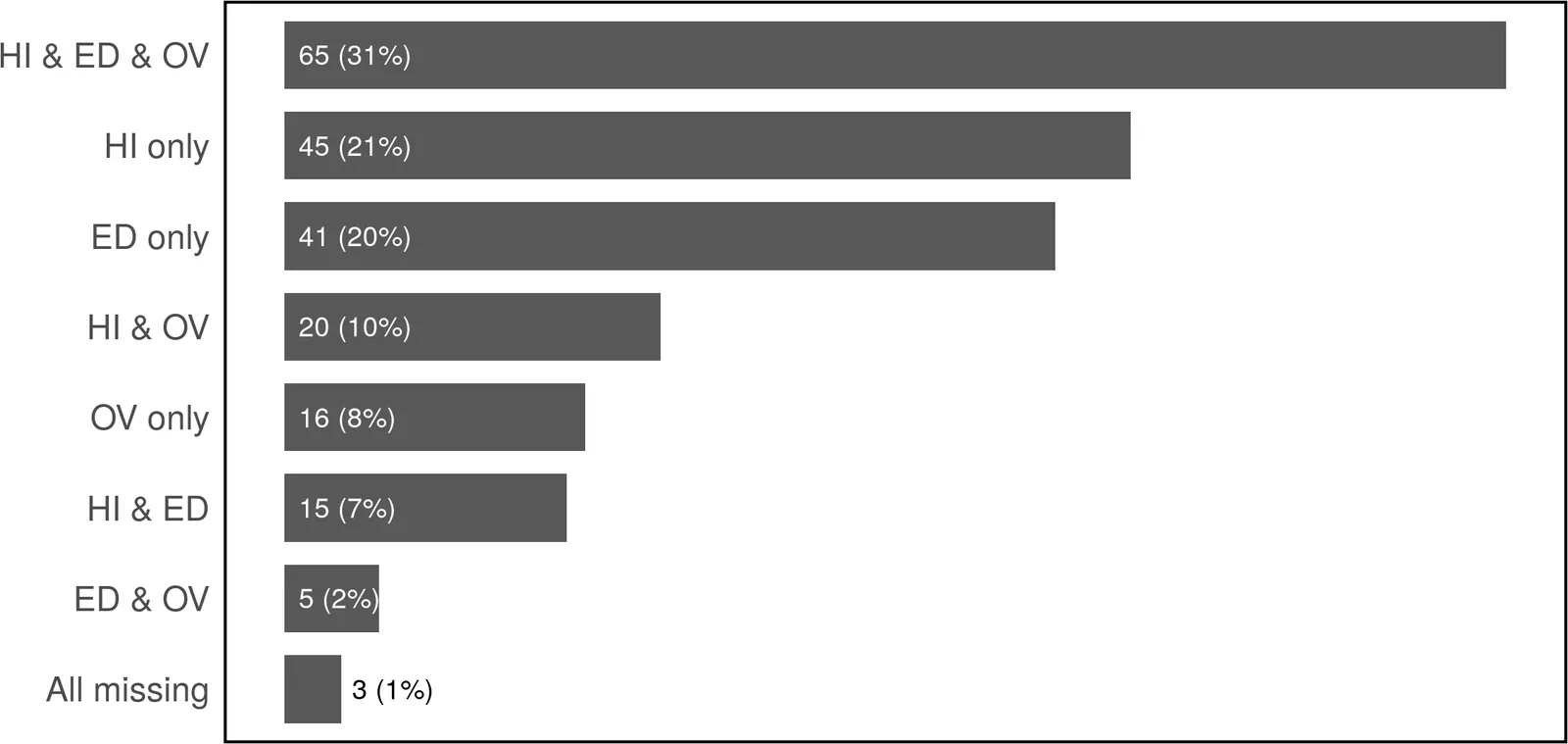
Locations where ASApp was used. Providers could select more than one location and this is represented by “&”. ED, emergency department, HI, hospital inpatient, OV, outpatient or clinic visit.

Survey data for each site was found to be similar and therefore combined for the analysis. (Supplementary material). Of 211 surveys, one was excluded due to missing responses to the primary outcome question, leaving 210 surveys answered by 97 (46%) staff physicians, 72 (34%) resident physicians, 23 (11%) pharmacists, and 17 (8,1%) nurses, Of staff physicians, 35 (36%), 25 (26%) and 36 (38%) respectively had been in practice 0–5, 6–15 and >15 years, respectively. Overall, 48% reported using the ASAapp both for inpatients and outpatients, 21% only in inpatients and 30% exclusively in outpatient settings (1 missing). (Figure 1)

Overall, 164/210 (78%), 162/209 (78%), and 150/210 (71%) self-reported using the ASApp recommendations for choice, dose and durations of antimicrobials, respectively, almost always or often. (see Supplementary Material) The perceived practice impact of the ASApp in various domains is seen in Table 1. Notably, 6 (3%) respondents reported no impact on practice. The information sections on clinical and diagnostic considerations were reported as helpful by 178 (85%) respondents.

**Table 1.** Self-reported impact on clinical practice with use of ASApp Table 1 long description.

Self-reported Impact of ASApp on clinical practice	N = 210 (%)
Choosing optimal antimicrobial agents	187 (89)
Aids in aligning practice with current Canadian guidelines	158 (75)
Supports duration of therapy decisions	156 (74)
Helps to determine weight/age-appropriate dosing	153 (73)
Provision of useful diagnostic guidance	138 (66)
Has not impacted my clinical practice	6 (3)
Other	3 (1)

## Discussion

Our analytics revealed that the ASApp was widely accessed (relative to the combined 242 medical beds at both hospitals) with a monthly average of 1658 unique users and 4,922 sessions over the prior 24-month period. A systematic review reported studies with fewer numbers of sessions, but many had shorter implementation times and were mainly focused on inpatients.^4^ Our survey revealed use of the ASApp in all care settings with 30% reporting use only in outpatient settings (ED and outpatient visits). Additionally, analytics revealed that the most frequent guidelines accessed are for typical outpatient infections. Together, the analytic and survey data combined suggest that our ASApp was used not only inpatients but importantly, in outpatient settings.

Although survey respondents largely reported using the ASApp recommendations for choice (78%) and durations (71%) and reported high impact in these domains, this does not imply recommendation adherence since prescribing data related to use of the ASApp could not be collected. Importantly, the gap between what is recommended and what is prescribed was recently highlighted in a study of pediatric outpatients where less than one third of prescriptions met the criteria for recommended choice and duration.^5^ Other studies, using similar self-assessment surveys reported improved guideline adherence after use of a mobile application.^6,7^ A report from a New Zealand pediatric hospital, however, did find appropriate antimicrobial prescriptions increased from 69% to 84% after an application was introduced whereas a study in Europe did not find a difference in overall appropriate antimicrobial prescribing 12 months following the introduction of an application for hospitalized adults.^8,9^

Remarkably, 97% of respondents reported an impact on practice in this survey compared with 41% reporting an impact in a hospital-based study.^10^ It is possible that the high reported impact is due to it’s educational value (85% reported that sections of clinical and diagnostic considerations are helpful) and existence for 8 years. Similar survey responses from the 2 geographically different site ASApps suggests that users likely trusted local guidance and adds to the generalizability of the results.

## Limitations

We are unable to determine whether the survey respondents are representative of the population of users. Survey respondents were biased toward more trainees (34%) and likely hospital-based physicians who are familiar with mobile applications and AS teaching thereby increasing acceptance. No prescription data was available for analysis as the ASApp is not linked to patient prescriptions therefore actual changes in practice cannot be verified. The survey analyses were descriptive only, precluding inferential or multivariable assessment of associations.

## Conclusions

Analytics of the ASApp from 2 small to medium-sized pediatric hospital sites revealed high numbers of users and sessions. At least one third of survey respondents reported use in outpatient settings, a known AS gap. Although the majority of users self-reported using recommendations and an impact on practice, corroboration with actual prescriptions was not available thereby limiting conclusions as to the actual impact. Future research priorities should prioritize determining changes in antimicrobial prescribing related to the use of AS mobile applications, especially in geographic areas or settings where there is a known stewardship gap.

## Supporting information

10.1017/ash.2026.10813.sm001 Le Saux et al. supplementary material 1 Le Saux et al. supplementary material

10.1017/ash.2026.10813.sm002 Le Saux et al. supplementary material 2 Le Saux et al. supplementary material

## Supplementary material

The supplementary material for this article can be found at https://doi.org/10.1017/ash.2026.10813.

## Table 1. Long description

A table titled ‘Self-reported Impact of ASApp on clinical practice’ with 210 respondents. The table has two columns: one for the impact category and one for the percentage of respondents. The impact categories include choosing optimal antimicrobial agents, aligning practice with current Canadian guidelines, supporting duration of therapy decisions, helping to determine weight/age-appropriate dosing, provision of useful diagnostic guidance, no impact on clinical practice, and other. The percentages are 89 percent, 75 percent, 74 percent, 73 percent, 66 percent, 3 percent, and 1 percent respectively. The table lists the number of respondents and the corresponding percentage for each impact category.

Navigate back to Table 1..
