# Supplementary material for: Does a mobile application lend a hand for promoting antimicrobial stewardship? A survey of users
Source: Antimicrob Steward Healthc Epidemiol. 2026 Sep 3;6(1):e254. doi: 10.1017/ash.2026.10813 (PMC13760412; doi:10.1017/ash.2026.10813)
Supplement: Supplementary file 1 [file S2732494X26108134sup001.docx]

Supplementary Material:

**Appendix 1 – Survey tool**

a. In which part of Canada do you primarily practice?
   a. Alberta
   b. British Columbia
   c. Manitoba
   d. New Brunswick
   e. Newfoundland and Labrador
   f. Northwest Territories
   g. Nova Scotia
   h. Nunavut
   i. Ontario
   j. Prince Edward Island
   k. Quebec
   l. Saskatchewan
   m. Yukon
b. Your role:
   1. Resident Physician
   2. Staff Physician
   3. Independent Nurse prescriber
   4. Pharmacist
   5. Nurse
   6. Other
c. How many years have you been in practice?
a. Currently still in training
b. 0-5 years
c. 6-10 years
d. 11-15 years
e. > 15 years
d. Where do you use the app? (*check all that apply)*
   a. Hospital inpatient
   b. Emergency Department
   c. Outpatient clinic or office setting

**Point of care decisions regarding antibiotic therapy**

e. How frequently do you use the Firstline app in making decisions about the following?

|  | Almost never | Once in a while | Sometimes | Often | Almost always | Not applicable |
| --- | --- | --- | --- | --- | --- | --- |
| The choice of antibiotic therapy |  |  |  |  |  |  |
| The dosage of antibiotic therapy |  |  |  |  |  |  |
| The duration of antibiotic therapy |  |  |  |  |  |  |

f. How helpful do you find the sections “clinical/diagnostic considerations” and “most likely pathogens” in your point of care decisions (e.g. meeting criteria for condition such as UTI)?

a. Helpful to see as a learning tool and supports my clinical decisions now and in the future.
b. Not helpful as a learning tool or in my clinical decisions as I use this APP mainly for dosing antibiotics.
c. I do not use this section enough to comment.

**Usability and usefulness of the Firstline app**

g. How would you rate the ease of navigating between different sections of the app? (Select one option)

- I can navigate instantly with no or minimal effort
- I sometimes need to pause and think about where to find things
- I frequently struggle to find what I’m looking for
- I need substantial assistance to navigate the app

i. In what ways has the app impacted your clinical practice? (Select any or all that apply)

- Provides useful diagnostic guidance
- Aids in aligning my practice with current Canadian guidelines
- Helps me choose optimal antimicrobial agents
- Helps me determine weight/age-appropriate dosing
- Supports duration of therapy decisions
- Has not impacted my clinical practice
- Other (please specify)

ii. Is there any information regarding antimicrobial prescribing that we should include or modify inthe

Firstline app?

- No
- Yes

iii. If yes, please tell us more [open text field]

Thank you for taking the time to share your experience with the Firstline app. Your feedback will help us improve clinical decision support for all users. As a token of appreciation, please follow the link below to provide your email address to be eligible for a gift card (optional)

<https://redcap.cheori.org/surveys/?s=W4WLRHCPNEAKK74E>
